# Support Quality Moderates the Impact of Total Household Occupancy on Self-Care Neglect in Informal Caregivers

**DOI:** 10.1093/geroni/igab046.1455

**Published:** 2021-12-17

**Authors:** Austin Matus, Barbara Riegel

**Affiliations:** University of Pennsylvania, Philadelphia, Pennsylvania, United States

## Abstract

Caregiver self-care may be impacted by the household environment. We evaluated the impact of support quality (e.g., ratings of quality of emotional support, information, material help, errands performed by others) and total household occupancy on a validated measure of self-care neglect in caregivers of patients with heart failure. Multivariate regression modeling was used to examine predictors of self-care neglect and we introduced an interaction term between support quality and household occupancy. The main effects model included terms for years of caregiving experience, hours caregiving daily, support quality, and total household occupancy (R2: 0.31; p <0.05). The interaction term between support quality and household occupancy contributed significantly (p < .05) to the respecified model (R2: 0.41; p <0.05). We suggest that the potential benefit of total household occupancy on caregiver self-care depends on perceived support quality. Clinicians should assess quality of household resources with caregivers during interactions.

